# Structural and functional analysis of a bile salt hydrolase from the bison microbiome

**DOI:** 10.1016/j.jbc.2024.107769

**Published:** 2024-09-12

**Authors:** Radwa Asar, Poonam Dhindwal, Antonio Ruzzini

**Affiliations:** 1 Department of Veterinary Microbiology, Western College of Veterinary Medicine, University of Saskatchewan, Saskatoon, SK, Canada; 2 Department of Biochemistry, Microbiology and Immunology, College of Medicine, University of Saskatchewan, Saskatoon, SK, Canada

**Keywords:** hydrolase, bile acid, bacteria, enzyme structure, enzyme kinetics, enzyme inhibitor

## Abstract

The bile salt hydrolases (BSHs) are significant constituents of animal microbiomes. An evolving appreciation of their roles in health and disease has established them as targets of pharmacological inhibition. These bacterial enzymes belong to the N-terminal nucleophile superfamily and are best known to catalyze the deconjugation of glycine or taurine from bile salts to release bile acid substrates for transformation and or metabolism in the gastrointestinal tract. Here, we identify and describe the BSH from a common member of the Plains bison microbiome, *Arthrobacter citreus* (BSH_Ac_). Steady-state kinetic analyses demonstrated that BSH_Ac_ is a broad-spectrum hydrolase with a preference for glycine-conjugates and deoxycholic acid (DCA). Second-order rate constants (*k*_cat_/*K*_M_) for BSH_Ac_-catalyzed reactions of relevant bile salts—glyco- and tauro-conjugates of cholic acid and DCA— varied by ∼30-fold and measured between 1.4 × 10^5^ and 4.3 × 10^6^ M^-1^s^-1^. Interestingly, a pan-BSH inhibitor named AAA-10 acted as a slow irreversible inhibitor of BSH_Ac_ with a rate of inactivation (*k*_inact_) of ∼2 h^-1^ and a second order rate constant (*k*_inact_/*K*_I_) of ∼24 M^-1^s^-1^ for the process. Structural characterization of BSH_Ac_ reacted with AAA-10 showed covalent modification of the N-terminal cysteine nucleophile, providing molecular details for an enzyme-stabilized product formed from this mechanism-based inhibitor’s α-fluoromethyl ketone warhead. Structural comparison of the BSHs and BSH:inhibitor complexes highlighted the plasticity of the steroid-binding site, including a flexible loop that is variable across well-studied BSHs.

Bile salts, which are amino acid-conjugated bile acids, have a well-established role in digestion. Bacterial biotransformation products of bile salts within the gastrointestinal tract of animals are increasingly appreciated for their potential direct and indirect impacts on health, infectious and chronic disease (1, 2, 3, 4, 5). Accordingly, the identities and relative abundances of primary (host-derived) and secondary (bacterially-modified) bile acids have been linked to many biological processes that extend far beyond their well-established role in digestion. While primary bile acids vary across *Animalia*, most mammals produce the C_24_ bile acids cholic and chenodeoxycholic acid (CA and CDCA), which are generated from cholesterol in the liver (6). There, they are also conjugated to an amino acid: glycine or taurine. Secondary bile acids, for example, deoxy- and lithocholic acid (DCA and LCA) are generated from CA and CDCA through a series of bacterial enzymatic reactions. Individual secondary bile acids possess discrete and multifaceted biological activities, although the majority are transported back to the liver *via* the enterohepatic circulation system where they too are substrates for amino acid conjugation and reused for digestion. Thus, the typical recirculating pool of bile salts includes conjugates of both primary and secondary bile acids.

Bacterial access to bile acids is restricted by amino acid conjugation. Bile salt hydrolases (BSHs) catalyze the amino acid deconjugation reactions required to liberate free bile acids for subsequent and successive modifications and metabolism. The BSHs belong to the N-terminal nucleophile hydrolase (Ntn-hydrolase) superfamily, which is characterized by an αββα core structure that is subject to autoactivation. A cysteine (Cys2) acts as the N-terminal nucleophile, and an additional suite of residues have been identified that have either established or tentative roles in catalysis. These include backbone and sidechain contributors to the oxyanion hole that supports intermediate formation during hydrolysis as well as potential determinants of substrate selectivity (7, 8). Generally, the BSHs can be characterized as possessing either narrow or broad substrate specificities. Those with a narrow substrate range are defined by their preference for a specific amino acid conjugate or steroid hydroxylation pattern. For example, the BSH from *Bifidobacterium longum* has a broad substrate range (9) whereas *Lactobacillus gasseri* encode for two distinct BSHs that each display a differential preference for either glycine or taurine conjugates (8, 10). The BSH of *Bacteroidetes thetaiotaomicron* (colloquially *B. theta*) does not catalyze the hydrolysis of CA-based bile salts whereas glycine- and taurine-conjugates of other bile acids are substrates (11). In addition to the major components of bile, non-canonical bile salts that differ in their amino acids have recently been reported (12). Remarkably, these bile salts are the products of an amine N-acyl transferase activity assigned to the BSHs (13, 14). These bile acids are referred to as microbially-conjugated bile acids (MCBAs), and there is increasing evidence that conjugation is not restricted to amino acids (15, 16).

Since the BSHs initiate bile acid modification and metabolism in the gastrointestinal tract, there has been significant interest in developing specific inhibitors to study their impacts on host biology. A screen of 2240 bioactive compounds resulted in the discovery that caffeic acid phenethyl ester, riboflavin, and high concentrations of tetracyclines could inhibit the *Lactobacillus salivarius* BSH (17). These compounds, however, lack specificity. Consequently, non-antibiotic, covalent pan-BSH inhibitors based on bile acids bearing an α-fluoromethyl ketone (FMK) warhead were developed using a rational and targeted approach (18). A series of these FMK-containing inhibitors have been shown to inhibit detectable BSH activities in bacterial cultures at nanomolar concentrations with minimal off-target interactions and were modified to restrict their biological distribution to the gut in animals (18). Structure-activity relationship studies of these FMK warhead mechanism-based inhibitors have resulted in a second-generation pan-BSH inhibitor named AAA-10 that consists of an LCA scaffold that is sulfated at C3 of the steroid A-ring (19). Importantly, the use of AAA-10, and therefore BSH inhibition, protects against membrane damage and hepatic inflammation in a rodent model of cirrhosis (20). These well-defined and controlled efforts are likely to continue to bridge knowledge gaps associated with BSH function, animal and human health and disease.

Our current state of knowledge of the BSHs is generally limited to members that are found in the gastrointestinal tract of humans. The activities and inhibition thereof, however, are also being considered in agriculture as alternatives to antibiotics for the management of food-producing animals (21). Moreover, DCA and taurine were recently identified as putative biomarkers of beef production and carcass quality (22) and a recent metagenomic analysis of dairy cattle identified 439 unique BSHs that were distributed across taxonomically diverse bacteria living in the small and large intestine (23). We recently became interested in the apparent prevalence of *Arthrobacter* spp., members of the class *Actinomycetia*, found to be associated with bison (24). Specifically, *Arthrobacter* spp. are the third most frequently recovered aerobe from bison feces whereas bacterial genera typically described for their roles in bile salt metabolism (*e.g. Lactobacillus*, *Enteroccocus*, *Clostridium* spp.) were far less frequently isolated (24). At the genus level, *Arthrobacter* are known to for their capacities to metabolize CA (25, 26, 27) and other *Actinomycetia*, namely *Bifidobacteria* spp., are known to encode for BSHs (9, 23). Thus, we hypothesized that these frequently associated members of the bison microbiome were involved in bile acid metabolism. In this work, we identify and describe a BSH from *Arthrobacter citreus*, a bacterium that was isolated from a fecal sample obtained from a Plains bison (*Bison bison bison*) living in Alberta, Canada. Identification of a single *bsh* gene in the *A. citreus* genome preceded recombinant enzyme production and characterization. Steady-state kinetic analyses and pan-BSH inhibitor kinetic studies are reported and discussed in the context of complementary structural data obtained using single-crystal X-ray diffraction experiments.

## Results

### Isolation of *A. citreus* and identification of a BSH

To begin to study whether or not bison-associated *Arthrobacter* spp. participate bile salt metabolism, we isolated a strain from a Plains bison fecal sample. The animal belonged to a repopulation project in western Canada (28), and we named the organism *A. citreus* EINP1 based on geographical origin (Elk Island National Park; EINP1) and genetic information. The genome of *A. citreus* EINP1 is comprised of a single circular chromosome of 3.88 Mb with an average GC content of 65.3%. Taxonomic analysis using the 16S rRNA gene suggested annotation of the isolate as *A. citreus* based on an ∼99% nucleotide sequence identity to the *A. citreus* ATCC11624 gene. A single probable *bsh* gene was identified within the *A. citreus* EINP1 genome.

**Figure 1:**
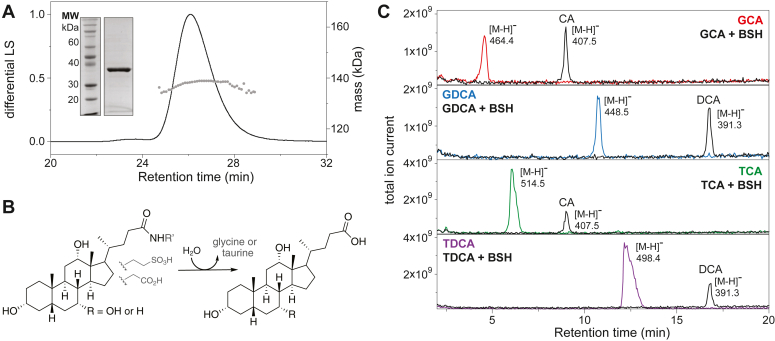
**Overview of BSH_Ac_ purification and activity.***A*, SDS-PAGE and SEC-MALS analysis of the recombinant BSH_Ac_ enzyme. *B*, The hydrolytic reaction catalyzed by BSHs. *C*, HPLC/MS experiments showing the BSH_Ac_-catalyzed deconjugation of four bile salts: GCA, GDCA, TCA and TDCA to CA or DCA. The observed substrate and product ions are labeled.

The *A. citreus* EINP1 BSH (BSH_Ac_) was produced and purified as a recombinant form. A C-terminally hexahistidine-tagged construct was prepared using *Escherichia* *coli* BL21(DE3) and immobilized metal affinity chromatography. The enzyme was purified as a tetramer with a molecular weight of 138 ± 2 kDa, measured by size-exclusion chromatography coupled to multiangle light scattering (SEC-MALS; Fig. 1*A*). We tested the ability of the BSH_Ac_ to deconjugate a series of biologically relevant bile salts, including amino acid conjugates of CA and DCA (Fig. 1*B*), which are the most abundant bile acids in adult bovids (23, 29). Overnight reactions of the BSH_Ac_ with glycine and taurine conjugates of CA and DCA (GCA, TCA, GDCA and TDCA) in 0.1 M MES, pH 6.0, resulted in the complete conversion of these substrates to deconjugated products, which could be readily detected by HPLC/MS (Fig. 1*C*). Overall, these results were considered functional annotation of the *A. citreus* enzyme as a BSH.

### Steady-state kinetic analysis of BSH_Ac_

**Figure 2:**
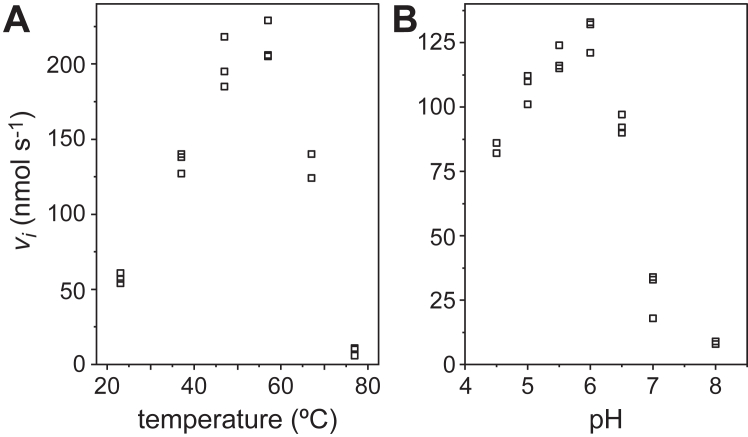
**Temperature and pH-dependent enzyme activity.***A*, temperature and *B*, pH dependence of BSH_Ac_ activity using 4 mM GDCA as the substrate.

**Figure 3:**
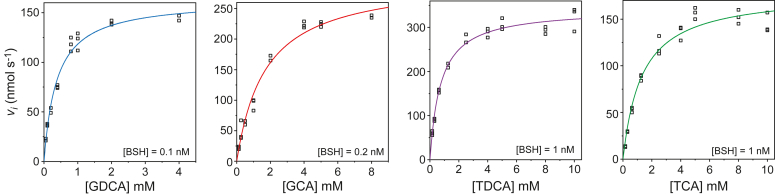
**Michaelis-Menten plots showing substrate-dependent *v***_**i**_**values for BSH**_**Ac**_**-catalyzed reactions.** Hydrolysis of GDCA, GCA, TDCA, and TCA shown *left* to *right*. Nonlinear fits to the Michaelis-Menten equation are shown as solid lines.

To gain insight into the specificities of BSH_Ac_-catalyzed reactions, we performed steady-state kinetic analyses using the four aforementioned physiological substrates. A discontinuous assay that relied on *o*-phthaladehyde to quantify liberated amino acid products at regular time intervals was employed to obtain the initial reaction velocities of BSH_Ac_-mediated deconjugation of GCA, TCA, GDCA and TDCA. Along with these steady-state analyses, the temperature and pH-dependence of the reaction between BSH_Ac_ and GDCA were measured (Figs. 2 and S1). Optimal BSH_Ac_ activity was observed near 55 °C and pH 6.0. While the buffer was varied for the pH-dependent measurements, temperature-dependent and all other steady-state kinetic analyses of BSH_Ac_ were performed in 0.1 M MES, pH 6.0, supplemented with 1 mM TCEP and 0.03% Brij-35 (v/v) at 37 °C. Under these conditions, the steady-state kinetic parameters for the reactions of BSH_Ac_ with the four bile salts revealed that both the substituents of the steroid ring and the identity of the amino acid were relevant (Figs. 3 and S2 and Table 1). The measured *K*_M_ values, which ranged between 0.38 to 1.7 mM, were ∼2- to 4-fold lower for DCA compared to CA conjugates. The measured *k*_cat_ values, which ranged between 180 to 1600 s^-1^, were ∼5- to 8-fold greater for the glycine than for the taurine conjugates. At 4.3 × 10^6^ M^-1^s^-1^, the reaction between BSH_Ac_ and GDCA was 5- to 31-fold more specific than the other reactions studied. The relative order of BSH_Ac_ substrate preference – determined by *k*_cat_/*K*_M_ – was GDCA > GCA > TDCA > TCA; however, this enzyme can accept a relatively broad range of bile salts as substrates.

**Table 1:** Steady-state kinetic parameters measured for BSH_Ac_

Substrate	*k*_cat_ (s^-1^)	*K*_M_ (mM)	*k*_cat_/*K*_M_ (M^-1^s^-1^)
GCA	1500 ± 50	1.7 ± 0.2	8.8 ± 0.1 × 10^5^
GDCA	1630 ± 40	0.38 ± 0.03	4.3 ± 0.4 × 10^6^
TCA	179 ± 5	1.3 ± 0.1	1.4 ± 0.1 × 10^5^
TDCA	342 ± 6	0.74 ± 0.06	4.6 ± 0.5 × 10^5^


Substrate	Inhibitor	*k*_inact_ (min^-1^)	*K*_I_ (μM)	*k*_inact_/*K*_I_ (M^-1^s^-1^)
GDCA	AAA-10	0.033 ± 0.001	23 ± 3	24 ± 3

### Time-dependent inactivation of the BSH_Ac_ by the pan-BSH inhibitor AAA-10

**Figure 4:**
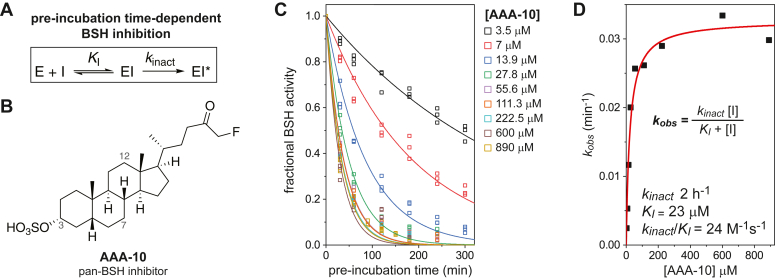
**Time-dependent inactivation of BSH_Ac_ by AAA-10.***A*, a steady-state kinetic scheme showing time-dependent inhibition of BSH_Ac_ by AAA-10. All species in the box may be present during the post-incubation reaction. *B*, the structure of AAA-10 is drawn with C3, C7, and C12 indicated. *C*, plot of BSH_Ac_ fractional hydrolase activity as a function of pre-incubation time with AAA-10. Fits for the rate of inactivation (*k*_obs_) at each concentration are shown as a solid line. *D*, plot of *k*_obs_ as a function of AAA-10 concentration and fit to the equation shown in panel *A* is shown (*red* line).

We next sought to assess the ability of the mechanism-based inhibitor AAA-10 to inactivate BSH_Ac_. Initial attempts to observe inhibition failed without pre-incubation of the enzyme and inhibitor (Fig. S3). Subsequently, time-dependent inhibition kinetic experiments were performed by pre-incubating BSH_Ac_ at 37 °C with varying concentrations of AAA-10 (Fig. 4). Pre-incubation of 200 nM BSH_Ac_ with AAA-10 for 30 to 300 min preceded the reaction of 0.1 nM of the mixture (containing enzyme and or enzyme:inhibitor complexes) with 4 mM GDCA. Initial reaction velocities were compared to uninhibited enzyme reactions, and these fractional BSH activities were observed to undergo a pre-incubation time-dependent exponential decrease (Fig. 4*C*). Thus, the kinetic behavior and data were characteristic of and fit to a two-step irreversible inhibition process in which the rate of inactivation is far slower than the formation of a non-covalent enzyme:inhibitor complex (Fig. 4*A*). In this scheme, the non-covalent enzyme inhibitor (EI) complexes that exist at any given pre-incubation time are rapidly disrupted upon dilution and mixing with excess substrate. The exponential rates of inactivation (*k*_obs_) observed across the concentration range of AAA-10 were used to determine the inactivation constant (*K*_I_) and rate of AAA-10 inactivation (*k*_inact_; Fig. 4, *C* and *D*). The *K*_I_ and *k*_inact_ were determined to be 23 ± 4 μM and 0.033 ± 0.001 min^-1^ (∼2 h^-1^), respectively. The inactivation efficiency (*k*_inact_/*K*_I_) of 24 ± 4 M^-1^s^-1^ was five orders of magnitude lower than the second-order rate constant determined for the reaction with GDCA (*k*_cat_/*K*_M_ ∼4.3 x 10^6^ M^-1^s^-1^), explaining the requisite pre-incubation to detect the activity of AAA-10 on BSH_Ac_.

### The three-dimensional structure of BSH_Ac_

**Figure 5:**
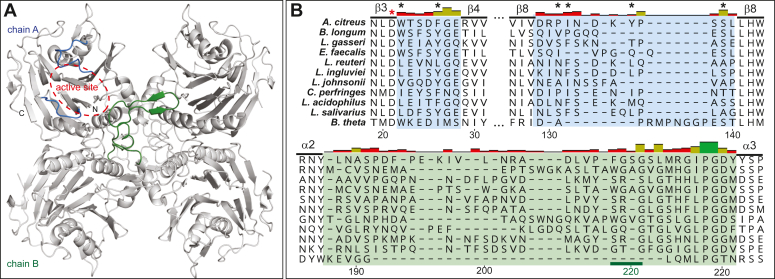
**The BSH_Ac_ tetramer, including relative positions and sequences of divergent active site loops.***A*, a cartoon representation of the BSH_Ac_ biological assembly. The loops of interest, which contribute to the active site, are colored in *blue* (chain A) and *green* (chain B) of the tetramer. The active site is outlined in chain A using a dashed *red* ellipse. *B*, sequence representation of three divergent loops of interest observed from a structure-based alignment of 11 BSHs. The *red* asterisk indicates a conserved active site residue (Asp21) and the *black* asterisks indicate steroid-binding residues. The solid *green* line spanning position 218 to 221 of the alignment highlights a previously assigned substrate selectivity motif.

To assess conserved and divergent structure-function relationships between BSH_Ac_ and other BSHs, we crystallized the enzyme and obtained a three-dimensional structure using X-ray diffraction data. A model of BSH_Ac_ was refined at a resolution 2.0 Å (Table 2). It showed the typical αββα-sandwich architecture and autoactivated N-terminal catalytic Cys (Cys2) nucleophile of the BSHs. The asymmetric unit of the BSH_Ac_ crystals contained a dimer. The protomers of this dimer are arranged in such a manner that part of the substrate binding site – the amino acid-binding region – is shaped by both protomers. In BSH_Ac_, a 36-amino acid loop extending from α3 and terminating in α4 contains a β-sheet as well as a proposed substrate selectivity motif (8). The latter participates in the formation of a dimer-dependent substrate-binding site with the loop swapped between the two protomers. At a higher level, this dimer-swapped (DS) loop also contributes to the tetrameric biological assembly of BSH_Ac_ (Fig. 5*A*). This tetrameric assembly was first described for the *B. longum* BSH (BSH_Bl_) as a dimer of dimers (7). In BSH_Ac_, the interface between the loop-swapped dimers covers an area of ∼2100 Å^2^ whereas the total area of interaction between protomers in the tetramer is ∼7200 Å^2^. The protomer interfaces include a series of hydrophilic and hydrophobic residues along with salt bridges involving residues Arg215, Asp220, Lys240, and Asp261. While the precise identities of many interface residues are poorly conserved in the tetramer, their overall characteristics are better conserved, and a few, namely Ile217, Gly219, His252, Glu272, and Tyr292 appear to be well- but not universally conserved across all the structurally-characterized BSHs (Figs. S4 and S5).

**Table 2:** Properties of BSH_Ac_ crystals, diffraction data, and refinement

Structure	BSH_Ac_	BSH_Ac_:AAA-10
PDB ID	8VRX	8VSY
Beamline	CLS, CMCF 08-ID	CLS, CMCF 08-ID
Wavelength	1.18	0.9537
Resolution range^a^ [Å]	36.14–2.04 (2.1–2.04)	42.95–1.6 (1.7–1.6)
Space group	C 1 2 1	C 1 2 1
Unit cell		
Cell dimensions		
a, b, c [Å]	167.8, 44.1, 86.6	167.8, 43.8, 87.2
α/β/γ (°)	90, 111.1, 90	90, 111.0, 90
Unique reflections^a^	37,834 (3741)	77,218 (7570)
Redundancy^a^	6.6 (6.6)	1.9 (2.0)
Completeness (%)^a^	99.7 (98.5)	98.2 (97.1)
I/σ (I)^a^	43.4 (20.2)	14.7 (4.8)
Wilson B-factor (Å^2^)	14.4	14.3
R_meas_^b^	0.036 (0.091)	0.041 (0.188)
Reflections used in refinement	37,828 (3741)	77,207 (7570)
R_work_/R_free_	0.13/0.17	0.14/0.16
CC_1/2_	1 (0.996)	0.998 (0.944)
Model contents (atoms)		
Non-hydrogen atoms	5735	5932
Macromolecules	5011	5070
Ligands	103	92
Solvent	621	770
Protein residues	633	638
R.M.S.D^c^		
Bonds (Å)	0.003	0.012
Angles (º)	0.62	1.5
Ramachandran plot		
Favored (%)	97.1	96.7
Allowed (%)	2.8	3.2
Outliers (%)		0.2
Average B factors [Å]		
Protein	13.6	15.8
All ligands	35.9	37.6
AAA-10^d^	N/A	19.9
Solvent	25.2	31.4

a The values in the parentheses are for the highest resolution shell.

b Indicates the redundancy independent merging R value.

c Root mean square deviation from restraint targets.

d AAA-10 was modeled at full occupancy.

The active site of the BSHs is defined by a series of conserved amino acids. This includes the N-terminal Cys nucleophile as well as Arg18, Asp21, Asn81, and Asn173. Specifically, the Asn81 backbone and Asn173 sidechain create an oxyanion hole that stabilizes tetrahedral oxyanion intermediates formed during catalysis. An acyl-enzyme mechanism was first proposed for the penicillin acylases of the Ntn hydrolase superfamily (30), and can be generalized for the BSHs. A first tetrahedral oxyanion intermediate is formed during the nucleophilic attack of the substrate’s amide bond by Cys2, which collapses to an acyl-enzyme to release an amino acid. Subsequently, the acyl-enzyme is attacked by water to form a second tetrahedral oxyanion intermediate that collapses to a bile acid and free enzyme. In the BSHs, the guanidinium group of Arg18 is positioned towards the Cys2 thiol(ate) and Asp21 is observed to H-bond with the Cys2 backbone oxygen. These residues thereby contribute to the reactivity of the N-terminal nucleophile and stabilize the charge throughout catalysis.

In addition to the conserved catalytic residues, the BSHs possess many contributors to substrate binding. Adjacent to the catalytic center, an amino acid binding pocket is formed, in part, by the DS loop as well as Arg226, Glu272, and Gln174. The binding of the steroid rings involves a relatively large number of hydrophobic residues: the substrate binding pocket of BSH_Ac_ is comprised of Trp22, Phe26, Ile58, Leu65, Phe67, Gly79, Leu80, Phe102, Pro131, Ile132, and Tyr136 through Ser139. Interestingly, the general character rather than the identities of amino acids expected to participate in substrate binding are conserved across the BSHs. The structural superposition of 11 BSHs that share between 22% and 57% sequence identity to BSH_Ac_ revealed a series of divergent features within loops inserted into the αββα sandwich core (Figs. 5*B* and S4 and Table S1). Specifically, low sequence identities and spatial divergence were noted in the loops that join β2 to β3, β8 to β9, and the DS loop connecting α3 and α4. Again, the latter is considered to be a substrate selectivity determinant; however, the BSH_Ac_ motif (GSG; Table S1) and observed substrate specificity oppose the structure-based prediction that GXG motifs prefer taurine conjugates (8).

### The three-dimensional structure of a covalent BSHAc:AAA-10 complex

**Figure 6:**
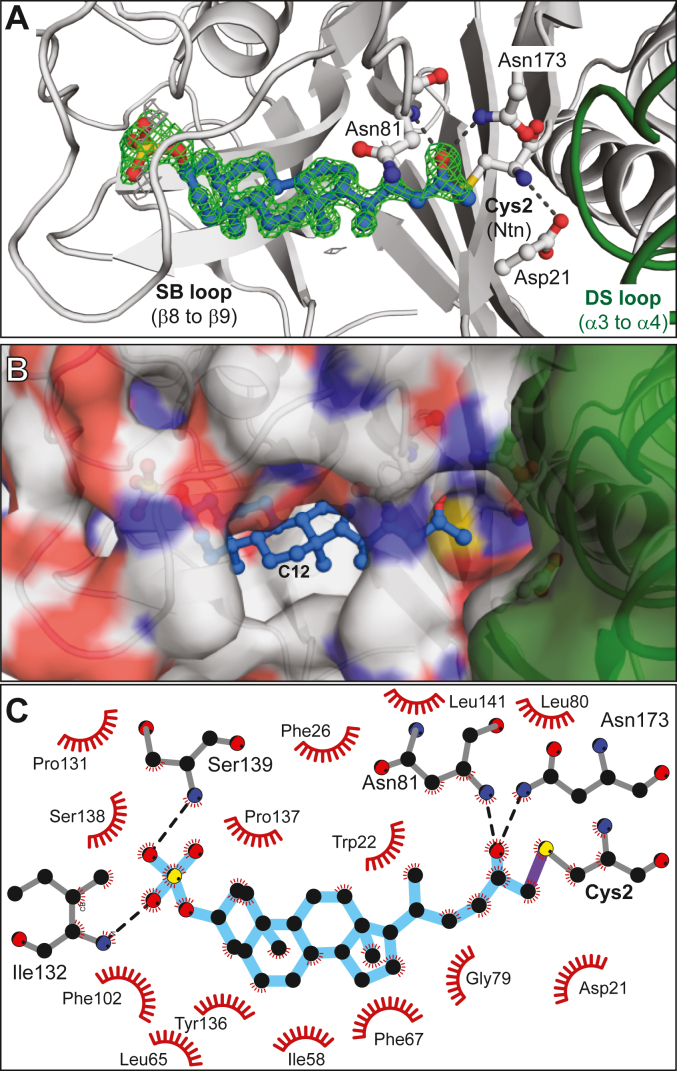
**Covalent inhibition of BSH_Ac_ by AAA-10.***A*, the active site of the BSH_Ac_:AAA-10 complex. Ball-and-stick representations of the inhibitor (*blue*) and key amino acids (*white*) are shown along with the unbiased (*F*_o_ – *F*_c_) density of the acyl-enzyme contoured at 3σ (*green*). *B*, a surface representation of the BSH:AAA-10 complex showing solvent exposure of the C-ring, in particular, C12, and the adjacent substrate-binding pocket formed by both protomers. *C*, a two-dimensional representation of BSH:AAA-10 interactions. The inhibitor is shown in *blue* and the C-S bond is drawn in *purple*. H-bond are shown as *black* dotted lines. The *red* spoked arcs show non-covalent contacts between the protein and inhibitor.

**Figure 7:**
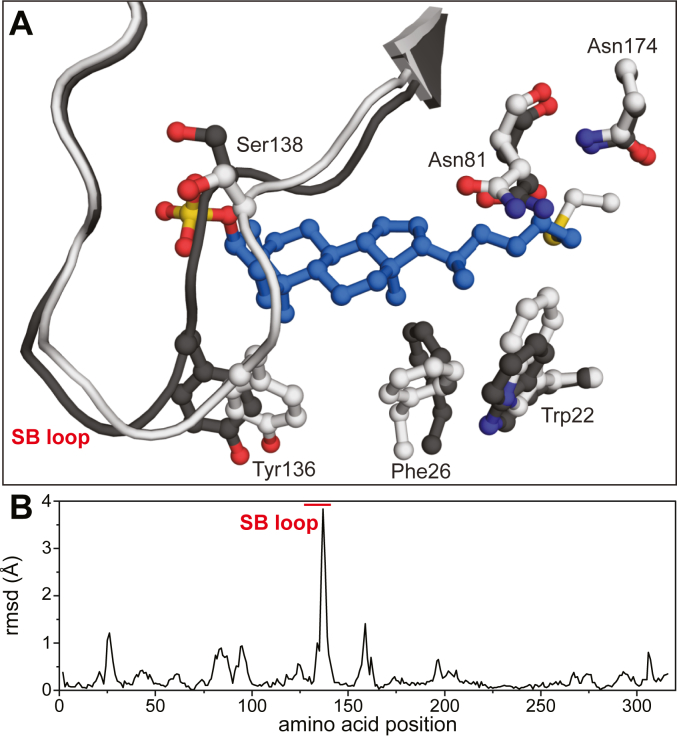
**Comparison of the empty and covalently modified BSH**_**Ac**_**active sites**. *A*, cartoon and ball-and-stick models showing changes to the position of the substrate binding (SB) loop and selected aromatic residues in unbound structure (*dark grey*) and AAA-10 bound structure (*white*). *B*, plot of the RMSD in Cα positions between unbound and bound BSH_Ac_.

To better understand the interactions between BSH_Ac_ and AAA-10, we crystallized the enzyme after soaking it with the inhibitor for 18 h in 0.1 M MES, pH 6.0 at room temperature. X-ray diffraction data were collected that resulted in the refinement of 1.6 Å resolution model showing a covalent adduct (Table 2). In the BSH_Ac_:AAA10 structure, electron density extending from Cys2 and the presence of defluorinated product resulting from sulfhydryl attack at the Cα of the FMK warhead was apparent (Fig. 6*A*). The inhibitor was modeled at full occupancy with the BSH oxyanion hole accommodating the product’s ketone oxygen atom. C12 of the LCA-based AAA-10 was notably solvent-exposed (Fig. 6*B*) and a series of polar and non-polar interactions appear to stabilize the inhibitor structure (Fig. 6*C*). The latter includes a group of hydrophobic amino acid residues within 4 Å of the inhibitor’s steroid core. Leu80, Asp21, Gly79, Phe102, and Leu141, for example, are well-conserved across BSHs. Additional BSH_Ac_ residues such as Ile58, Leu65, Phe67, Phe102, Ile132, and Tyr136 are generally not conserved but replaced by other non-polar amino acids in homologs. Inhibitor binding perturbs the position of a number of these amino acids relative to those observed in the free enzyme. In the inhibitor-free BSH_Ac_, residues Trp22 and Phe26 appear in a T-shaped π-stacking arrangement whereas the presence of the inhibitor alters their interaction. Specifically, Trp22, Phe26, and the nearby Tyr136 shift to accommodate the inhibitor, enclosing it from solvent. Finally, two of the C3-sulfate oxygens interact with the backbone amides of Ile132 and Ser139, and a series of water molecules are also present in the inhibitor-occupied active site. Again, a comparison of unbound and bound BSH_Ac_ active sites, reveals substantial differences in the positioning of a loop carrying both Ile132 and Ser139. We refer to this loop, which is located at the distal edge of the substrate binding site as the substrate-binding (SB) loop (Fig. 7*A*). The position of the atoms in the SB loop differs significantly between unbound and AAA-10 bound BSH_Ac_ models (Fig. 7*B*). While the apparent flexibility of this loop allows for the C3-SO_4_ group of AAA-10 to bind, there is clear displacement of residues that are likely to have roles in stabilizing a substrate. Across the BSHs, this loop is variable in length and sequence identity (Fig. 5*B*), and it appears to be quite flexible, supporting a role in substrate binding (Fig. S6).

## Discussion

This study identifies the *A. citreus* BSH as a possible contributor to bile salt metabolism in Plains bison. The substrate specificities for the reactions of BSH_Ac_ and the four bile salts evaluated are on the same orders of magnitude (*k*_cat_/*K*_M_ ∼10^5^ to 10^6^ M^-1^s^-1^) as those reported for the well-studied BSHs of *B. longum*, *Clostridium perfrigens*, *Lactobacillus plantarum* and *Enterococcus faecalis* (7, 31, 32). Moreover, our results provide a better understanding of how a recently developed class of pan-BSH covalent inhibitors act on enzymes that are divergent from those used for inhibitor design, discovery, and development (18). Overall, the study highlights how mechanism-based inhibition of BSHs might vary between divergent homologs that occupy the same or similar niches.

Our kinetic analysis of AAA-10 represents the only such effort to date. Prior studies of disparate BSHs have suggested that rates of inactivation and potency of this class of inhibitor were orders of magnitude faster and greater than what was observed for BSH_Ac_. For example, the treatment of the *B. thetaiotaomicron* BSH with 100 μM AAA-1 (initially named compound 7), which is an FMK-bearing, non-sulfonated urseodeoxycholic acid, was reported to abolish detectable enzyme activity within 15 s (18). And, inhibition of BSH activity in cultures of *B. thetaiotaomicron* and *B. longum* by AAA-10 and AAA-2 (a C3-sulfonated form of AAA-1) were characterized by IC_50_ values between 74 and 901 nM (18, 19). We hypothesize that the disparity between these measures and our kinetic analyses is related to the divergence of BSH_Ac_ from the aforementioned enzymes. The relatively slow inactivation of BSH_Ac_ by AAA-10 suggests that reaction-competent positioning of the compound is limiting. The *k*_inact_/*K*_I_ for the reaction of BSH_Ac_ and AAA-10 was ∼24 M^-1^s^-1^, orders of magnitude lower than *k*_cat_/*K*_M_ values measured for reactions with substrates (10^5^ to 10^6^ M^-1^s^-1^). No inhibition of BSH_Ac_ by AAA-10 could be detected without pre-incubation. Both slow- and fast-acting FMK warhead-containing inhibitors have been previously reported, including second-order rate constants determined for other enzyme families that employ a cysteine nucleophile. Irreversible inhibition of the hepatitis A virus 3C cysteine proteinase by a peptidyl-FMK inhibitor was measured at 330 M^-1^s^-1^ (33) and values between 290 to 280,000 M^-1^s^-1^ were measured for peptidyl-FMK inhibition of a number of caspases (34). Our results and previous studies highlight the differential effects of pan-enzyme class inhibitors. Demonstration of inhibition of BSH_Ac_ by AAA-10 also calls attention to the breadth of the inhibitor’s target diversity. In dairy cattle, a well-studied and close relative of bison, for example, >400 unique BSHs (or AAA inhibitor targets) have been identified using a metagenomic approach (23). Accordingly, chemical proteomic approaches will be welcomed to help resolve differential effects and targets within the complex bacterial communities that colonize the gastrointestinal tracts of mammals.

The BSH_Ac_:AAA-10 complex structure is one of several BSH:inhibitor/chemical probe complexes available [(18), Table S1]. A comparison of BSH_Ac_ and BSH_Bl_ is informative as they possess some remarkable differences in their active sites despite sharing relatively high overall sequence identity (57%). The SB loop is poorly conserved with little conservation between these two homologs, let alone more divergent enzymes (Fig. 5*B*). In addition, this distal loop is commonly omitted from structural models of BSHs (Fig. S6). Across the available BSH:AAA-series complex structures, significant variability in its position is observed, highlighting flexibility to accommodate gut-restricted inhibitors and chemical probes. While this can be interpreted as active site plasticity, there are clearly limits. Accordingly, this SB loop of BSH_Ac_ may impede rather than accommodate inhibitor binding. This interpretation helps to explain the significantly lower-than-anticipated second-order rate constant for BSH_Ac_ inactivation. Therefore, this loop – variations therein – should be taken into account during future BSH inhibitor and chemical probe development. In contrast to this divergent feature of the BSHs, the BSH_Ac_:AAA-10 complex structure and others (Fig. S5) show that modifications to the C12 position (*e.g.* the C12 hydroxyl of a DCA- or CA-based inhibitor) should be tolerated based on exposure of this region to the bulk solvent. We expect considerable insight from continuing to evaluate the structure-function relationships of diverse BSHs with the AAA series compounds.

BSH substrate preference is clearly multifactorial, and our understanding of it is incomplete. Our study of BSH_Ac_ challenges the interpretation of prior structure-function relationship studies. The substrate specificity profile of BSH_Ac_ deviated from expectation based on the recently defined substrate specificity loop of homologs from the Family *Lactobacilliaceae* (8). More explicitly, two divergent motifs, GXG or SRX, found on the domain-swapped active site loop were assigned as determinants for either taurine or glycine-conjugated bile acids, respectively. BSH_Ac_, however, possesses a GSG motif and shows a preference for glycine rather than taurine-conjugates. Indeed, there is substantial divergence in the substrate binding sites of even the handful of biochemically- and biophysically-characterized BSHs. While this large solvent-exposed site possesses generalizable characteristics such as the presence of hydrophobic residues to facilitate steroid binding, studies of how these residues impact catalysis are scarce. The importance of electrostatics, van der Waals interactions, and steroid hydroxylation have recently been considered for the *L. salivarius* BSH (35). In light of the newly discovered BSH *N*-acyltransferase activities (13, 14), robust analyses of how the steroid-binding site influences catalysis should not be overlooked. Similarly, residues that impact hydrolysis of MCBAs should be investigated. For example, the Arg of the SRX motif has been assigned a role in the hydrolysis of MCBAs: a cation-π interaction was proposed to impart specificity for tyrosine and phenylalanine-conjugated CA (32). Understanding the determinants of BSH catalysis whether it is hydrolysis or *N*-acyltransfer will be critical to inform the potential therapeutic use of pan- or perhaps selective inhibitors in the future. More comprehensive mutagenesis, structure-function and *in vitro* and *in vivo* inhibition studies are needed to identify and characterize important determinants for all the reported BSH-mediated reactions. Ultimately, these studies will help disentangle the roles of BSHs and their impact on animal health and disease.

## Experimental procedures

### Isolation and genetic characterization *A. citreus*

*A. citreus* EINP1 was isolated from a fecal sample from a Plains bison being transported from Elk Island National Park (EINP) to Banff National Park in Alberta, Canada, in 2018 (obtained with permission, Parks Canada Permit no. EI-2018-29,479). The bacterium was one of many recovered using non-selective R2A agar supplemented with 200 μg/ml cycloheximide. Initial identification was accomplished through 16S rRNA gene sequencing. For whole genome sequencing, DNA was extracted from a 200 ml overnight culture of *A. citreus* EINP1 grown in 1% tryptone (w/v). Briefly, the culture was harvested by centrifugation at 4000 *g* for 20 min and the cells were flash-frozen in liquid N_2_. Then, using a mortar and pestle, the frozen cell pellet was ground to a fine powder in liquid N_2_ before being resuspended in TE buffer (10 mM TRIS HCl, 1 mM EDTA, pH 8.0) supplemented with 2.5 mg/ml hen egg white lysozyme, 1 mg/ml Proteinase K from *Tritirachium album* and 1% SDS (w/v). Nucleic acids were precipitated using an equal volume of *iso*-propanol, treated with RNase A, and purified by extraction using phenol:chloroform:isoamyl alcohol (25:24:1) saturated with TE buffer. Long-read DNA sequencing was performed using the PacBio Sequel platform and *de novo* genome assembly was performed using the hierarchical genome assembly process (36). Taxonomic analysis was performed using blastn (37) and the 16S ribosomal RNA sequences (Bacteria and Archaea) database (38). The BSH was identified using RAST (39), and the corresponding NCBI Reference Sequence is WP_342023006.1.

### Recombinant BSH production and purification

A synthetic and *E. coli*-codon optimized version of the *A. citreus* bile salt hydrolase (*bsh*_*Ac*_) was cloned into a pET29b(+) vector using the NdeI and XhoI sites to encode for a C-terminal hexa-histidine tagged form of the enzyme (Twist Bioscience). *E. coli* BL21(DE3) was used as an expression host and the pET29:*bsh*_Ac_ vector was maintained by the presence of 50 μg/ml of kanamycin sulfate. For protein production, bacteria were initially grown at 37 °C with shaking at 200 rpm to an optical density at 600 nm between 0.5 and 0.6. Then, *bsh*_Ac_ expression was induced by the addition of 1 mM isopropyl-1-thio-D-galactopyranoside (IPTG), and the culture was transferred to a refrigerated incubator operating at 16 °C with shaking at 200 rpm overnight. Cells were harvested by centrifugation at 12,000 *g* for 10 min and stored at −80 °C until protein purification was performed. The resulting *E. coli* cells harboring BSH_Ac_ were thawed and biomass from 1 L of culture was resuspended in 40 ml phosphate-buffered saline (PBS), pH 7.4, supplemented with 10 μg/ml DNase, 2.5 mM MgCl_2_ and 2.5 mM CaCl_2_. Cells were disrupted using a high-pressure homogenizer at 25,000 PSI and the debris was removed by ultracentrifugation at 40,000 *g* at 4 °C for 1 h. The resulting supernatant was incubated with 5 ml Nickel-nitrilotriacetic acid (NiNTA) resin for 15 min at room temperature and poured into a glass column. The NiNTA resin was washed in using a stepwise protocol with 20 mM TRIS-Cl, pH 8.0 buffers containing increasing concentrations of imidazole until the BSH_Ac_ enzyme was eluted at 200 mM imidazole. High-purity BSH_Ac_-containing fractions (assessed by SDS-PAGE) were pooled and concentrated using a 10 kDA-cutoff centrifugal concentrator. Purified protein was flash-frozen in liquid nitrogen at concentrations >10 mg/ml and stored at −80 °C until assayed. The purification yield was ∼50 mg of BSH_Ac_ per litre of *E. coli* BL21(DE3) culture.

### SEC-MALS analysis

SEC-MALS of purified BSH_Ac_ was performed using a Bio-Rad FPLC system in tandem to Multi-Angle Light Scattering (MALS) system. Purified protein was diluted to a concentration of 2 mg/ml using PBS, pH 7.4, before injection onto a size exclusion column (SEC; Superdex 200 Increase 10/300 Gl; Cytiva) pre-equilibrated in the same buffer. MALS and refractive index (RI) data were measured using DAWN HELEOS II and Optilab T-rEX detectors (Wyatt Technology). The system was calibrated with 2 mg/ml of bovine serum albumin prior to measuring the molecular weight of the BSH_Ac_.

### BSH reaction monitoring by HPLC/MS

To assess bile salt hydrolase activity, a series of reactions were performed using 2.5 μM BSH_Ac_ and 1 mM GDCA, GCA, TDCA, and TCA in 0.1 M MES buffer, pH 6.0, at room temperature. The reaction mixtures were incubated at room temperature overnight before mixing with acetonitrile 7:3 (v/v), centrifuged for at 20,000 *g* for 5 min, filtered, and assessed by HPLC/MS. An Agilent Technologies 1260 Infinity II HPLC system coupled to an Advion Expression single quadrupole compact mass spectrometer using an electrospray ionization source was employed. Separation of bile salts and acids was achieved using a Phenomenex Luna C18 column (100 × 4.6 mm, 5 μm, 100 Å). A two-solvent system was employed: solvent A was comprised of 0.05% formic acid in water and solvent B was comprised on acetonitrile/0.05% formic acid. After 3 min at 40% solvent B, the bile salts and acids were eluted using a gradient to 70% solvent B in 20 min. Negative ions of eluted compounds were detected between 100 and 2000 m/z.

### Enzyme activity measurements

BSH_Ac_ activity measurements were performed using a discontinuous assay that relied on the reaction of *o*-phthaladehyde (OPA) with the primary amine of the amino acid products of the deconjugation reaction. All reactions were conducted in the presence of a reducing agent 1 mM tris(2-carboxyethyl)phosphine (TCEP) and 0.03% Brij-35 (w/v). The buffer was varied for pH-dependent studies: 0.1 M Na-acetate at pH 4.5 and 5.0, 0.1 M MES at pH 5.5 and 6.0, 0.1 M potassium phosphate at pH 6.5 and 7.0 and 0.1 M HEPES at pH 8.0. For all steady-state kinetic measurements 0.1 M MES, pH 6.0, supplemented with 1 mM TCEP and 0.03% Brij-35 (w/v) was employed, and reactions were performed at 37 °C using a temperature-controlled water bath. The concentrations of GCA, GDCA, TCA, and TDCA used in the steady-state kinetic experiments ranged between 0.1 to 10 mM with specific concentrations varying by substrate. The concentration of BSH_Ac_ used in each reaction was varied between 0.1 to 2 nM to ensure that substrate consumption was <10% throughout each experiment. The 0.1 M MES, pH 6.0, 1 mM TCEP, 0.03% Brij-35 buffer was also used to evaluate the reaction of BSH_Ac_ and 4 mM GDCA at different temperatures between 22 and 77 °C. For steady-state kinetic measurements, reactions were initiated through the addition of enzyme and were quenched after defined time intervals using NaOH (83.3 mM final). Initial reaction velocities (*v*_i_) were determined by linear fitting of reaction progress that was monitored at defined intervals (1–3 min) over time courses of 4 to 18 min. Prior to product detection, the pH of the quenched reaction mixtures was adjusted through the addition of an equimolar amount of glacial acetic acid. For product detection, the quenched and pH-adjusted solution was mixed with a 1.2 mM OPA solution in 100 mM sodium borate, pH 9.5, containing 1.2 mM 2-mercaptoethanol and 3.3% ethanol (v/v). Reactions with OPA were allowed to proceed for 20 to 30 min prior to performing fluorescence intensity measurements using a Varioskan LUX Multimode plate reader (ThermoFisher). Data was collected using excitation and emission wavelengths of 340 nm and 455 nm, respectively, at a 5 nm bandpass. In order to calculate product concentrations and accurately normalize experimental measurements, a standard curve using the amino acid product (glycine or taurine) was included within each experimental 96-well plate. Quantification of the products from reactions performed in triplicate were used to produce plots of *v*_i_ and their dependence on pH, temperature, and substrate concentration. For the latter, a Michealis-Menten model was fit to the data to determine *k*_cat_, *K*_M_ and *k*_cat_/*K*_M_ values.

To determine the kinetic parameters for enzyme inactivation, *K*_I_ and *k*_inact_, a pre-incubation approach was required to evaluate a slow irreversible process (40). BSH_Ac_ was pre-incubated with AAA-10 at concentrations between 3.9 and 890 μM at 37 °C in 0.1 M MES, 1 mM TCEP, 0.03% Brij-35 (w/v), pH 6.0 for time intervals spanning 30 to 300 min. After the defined pre-incubation period, the enzyme was diluted 2000-fold to 0.1 nM in a 4 mM GDCA solution prepared in the same buffer system. The initial reaction velocities were measured and normalized to those of control reactions in which BSH_Ac_ was subject to a matching pre-incubation period in the absence of AAA-10. Plots of fractional BSH_Ac_ reaction velocities as a function of pre-incubation time were fit to an exponential equation to determine first-order rate constants (*k*_obs_) at a given AAA-10 concentration. Plots of *k*_obs_ as a function of AAA-10 concentration were then used to determine *K*_I_ and *k*_inact_ using a model that assumes saturation (see equation in Fig. 4*D*).

### Crystallization, data collection, and structure refinement

BSH_Ac_ and the BSH_Ac_:AAA-10 complex were crystallized using the hanging drop vapor diffusion method at room temperature. Initial screening and optimization of crystal formation were performed using PEG3350 as a precipitant prepared at 20 to 24% w/v in 0.1 M HEPES, pH 7.3 to 7.5 supplemented with 0.2 M MgCl_2_. The highest diffraction quality crystals of BSH_Ac_ formed by mixing a solution at 5 mg/ml at a 1:1 ratio of reservoir solution comprised of 0.1 M HEPES pH 7.5, 22% PEG3350, 0.2 M MgCl_2_. To prepare BSH_Ac_:AAA-10 complexes, the enzyme (5 mg/ml) was reacted with 560 μM AAA-10 in 0.1 M MES, pH 6.0, for 18 h and diffraction quality crystals were obtained when mixed 1:1 with 0.1 M HEPES pH 7.6, 21% PEG3350, 0.2 M MgCl_2_ solution. In all cases, crystals were transferred to a cryoprotectant containing the respective reservoir solution supplemented with 10% glycerol (v/v), and then flash-frozen in liquid nitrogen.

Single crystals were diffracted under cryogenic conditions (100 K) at the Canadian Light Source (CLS) using a Canadian Macromolecular Crystallography Facility Insertion Device (CMCF-ID) beamline, which is equipped with a Pilatus3 S 6M X-ray detector. Diffraction images were scaled using an automatic processing pipeline MXpro (developed by the CLS and previously known as AutoProcess) that relies on XDS (41), POINTLESS (42), and BEST (43). The initial phases for both structures were obtained by molecular replacement using the BSH from *L. salivarius* [PDB ID: 5HKE (44)] as the search model. Iterative cycles of refinement using the phenix.refine and COOT, included in PHENIX software package (45, 46, 47, 48), produced the final models of BSH alone or covalently modified by AAA-10. The coordinates and CIF file directory for AAA-10 inhibitor were generated using SMILES obtained from PubChem and eLBOW included in PHENIX package (49). All pre-existing PDB data were downloaded from the RCSB Protein DataBank (50). Pairwise structural analysis and figures were generated using PyMol (https://pymol.org/support.html), Chimera (51), and LigPlot+ (52).

## Data availability

The complete genome assembly of *A. citreus* EINP1 and the DNA sequencing experiment have been made available through the NCBI (Genbank accession no. CP151657; SRR Accession no. SRR28875536). X-ray diffraction data has been deposited in the Protein Data Bank under the accession codes 8VSY and 8VRX.

## Supporting information

This article contains supporting information (7).

## Conflict of interest

The authors declare that they have no conflicts of interest with the contents of this article.
