# Supplementary material for: Structural and functional analysis of a bile salt hydrolase from the bison microbiome
Source: J Biol Chem. 2024 Sep 12;300(10):107769. doi: 10.1016/j.jbc.2024.107769 (PMC11736000; doi:10.1016/j.jbc.2024.107769)
Supplement: Supplementary Figures S1-S6 and Table S1 [file mmc1.pdf]

Supporting Information for

Structural and functional analysis of a bile salt hydrolase from the bison microbiome

Radwa Asar[1*], Poonam Dhindwal[1*] and Antonio Ruzzini[1,2,#]

[1]Department of Veterinary Microbiology, Western College of Veterinary Medicine, University of Saskatchewan, Saskatoon S7N 5B4

[2]Department of Biochemistry, Microbiology and Immunology, College of Medicine, University of Saskatchewan, Saskatoon, S7N 5E5

*these authors contributed equally to this work

#corresponding author

Figure S1. Reaction progress curves for temperature- and pH-dependent BSH_{Ac} activity S2
Figure S2. Steady-state kinetic reaction progress curves for BSH_{Ac}-catalyzed bile salt hydrolysis S3
Figure S3. BSH_{Ac} activity in the presence of AAA-10 without pre-incubation S4
Figure S4. Sequence alignment derived from structural superposition of 11 BSHs S5
Figure S5. Conserved protomer interface residues S6
Figure S6. Structural superposition of BSH_{Ac}:AAA-10 and other BSH:AAA series S7
Table S1. Overview of BSH and BSH:inhibitor structures available from the PDB S8

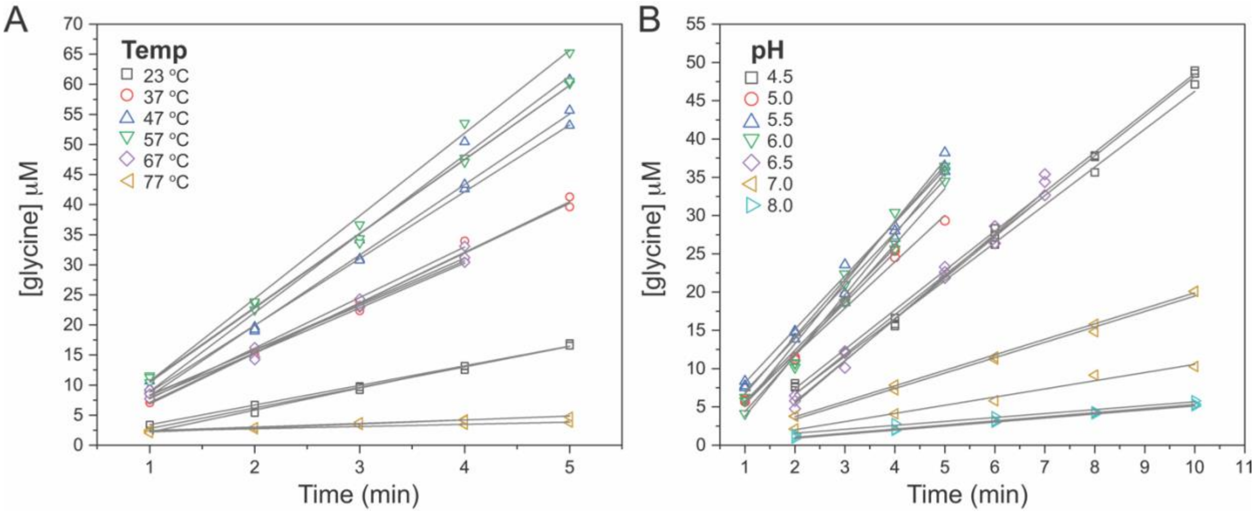

Figure S1. Reaction progress curves for temperature- and pH-dependent BSH$_{Ac}$ activity. The plots show time-dependent BSH$_{Ac}$-catalyzed hydrolysis of 4 mM GDCA. Quantification of glycine was used to determine the impact of **(A)** temperature and **(B)** pH on the rate of this reaction. Linear fits to the data are shown in grey.

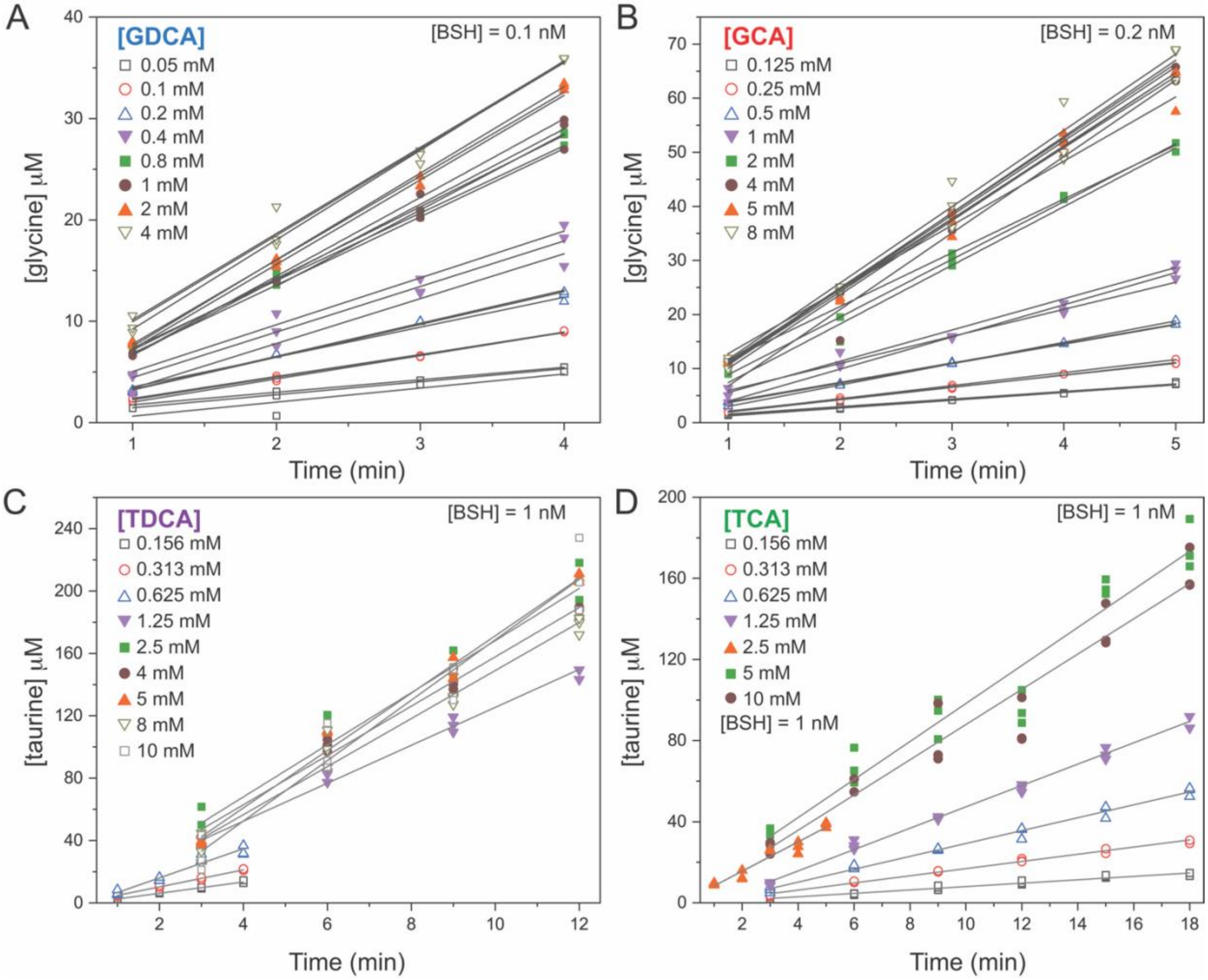

Figure S2. Steady-state kinetic reaction progress curves for BSH_{Ac}-catalyzed bile salt hydrolysis. The plots show time-dependent BSH_{Ac}-catalyzed amino acid hydrolysis. Quantification of free amino acids was used to determine initial reaction velocities for BSH_{Ac}-catalyzed deconjugation of four bile salts: **(A)** GDCA, **(B)** GCA, **(C)** TDCA and **(D)** TCA. Linear rates were observed and fit (grey lines) over shorter (*e.g.* 4-5 min) or longer (*e.g.* 12-18 min) periods for more- or less-specific reactions, respectively. The concentration of BSH_{Ac} was varied between 0.1 to 1 nM (indicated on this plot and in Figure 3) for reactions with distinct substrates.

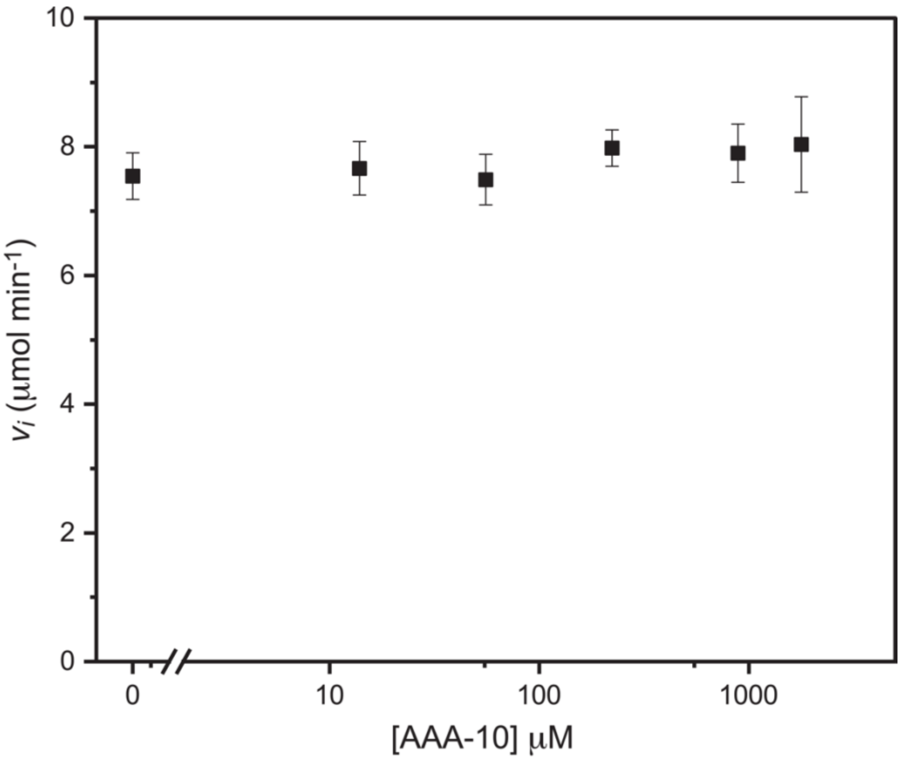

Figure S3. BSH_{Ac} activity in the presence of AAA-10 without pre-incubation. The plot shows the initial reaction velocities (v_i) when 0.1 nM BSH_{Ac} was reacted with 4 mM GDCA in 0.1 M MES, pH 6.0, supplemented with 1 mM TCEP and 0.03% Brij-35 (v/v) in the absence of inhibitor and in the presence of varying concentrations of AAA-10 (13.9, 55.6, 222.5, 890 and 1780 μM). Reactions were initiated through the addition of enzyme to solutions containing GDCA or GDCA and AAA-10.

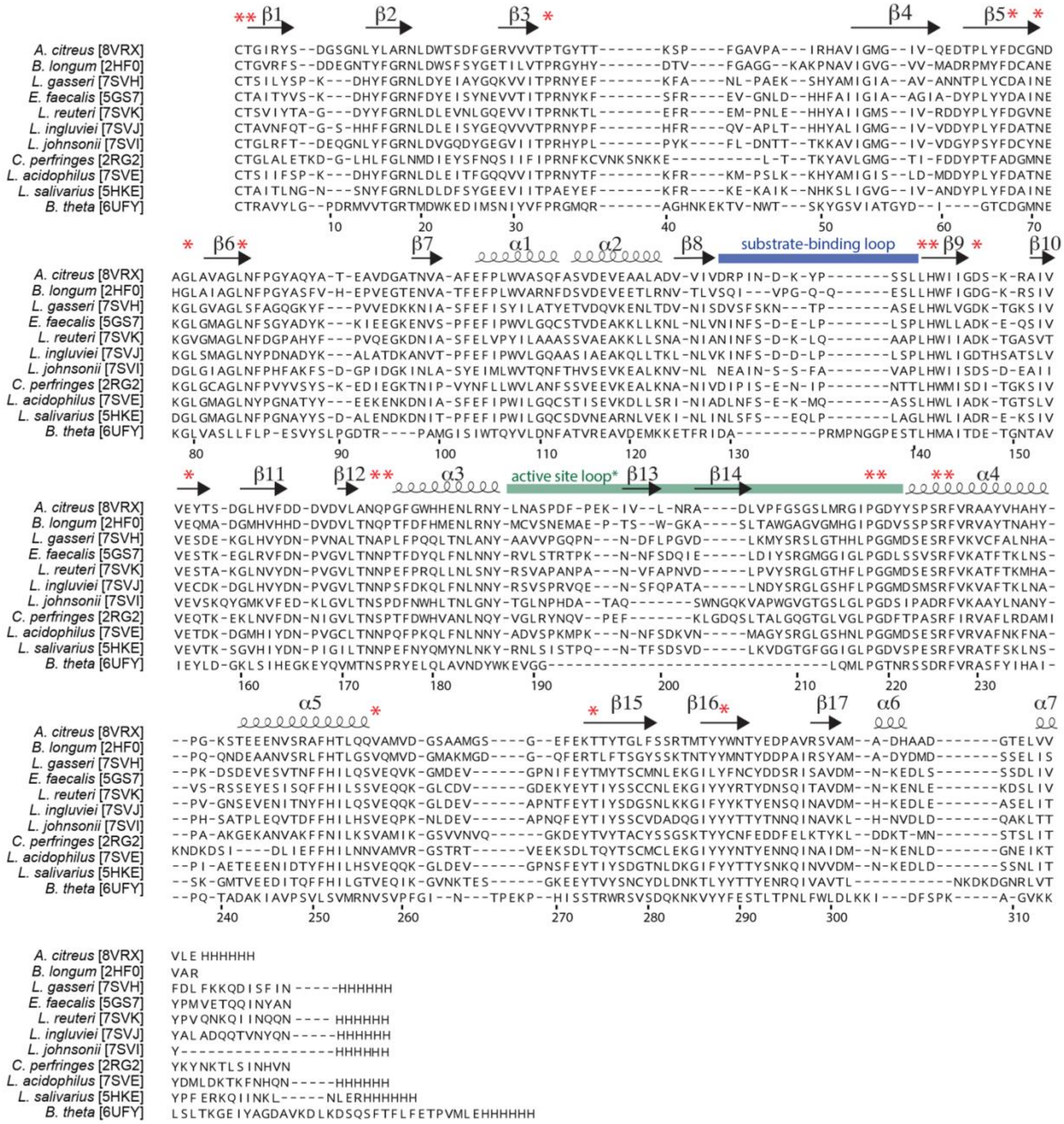

Figure S4. Sequence representation of the structural alignment of 11 BSHs. The secondary structural elements, conserved amino acids(*), and active site loops of interest are indicated. The substrate binding loop (SB loop), which interacts with the steroid and C3-SO_4 group of the AAA-10 inhibitor is highlighted in blue. The dimer protomer swapped active site loop (DS loop), which includes a small antiparallel β-sheet, is shown in green. The bacterial origin and structural data used for the analysis [PDB IDs] are listed at the left.

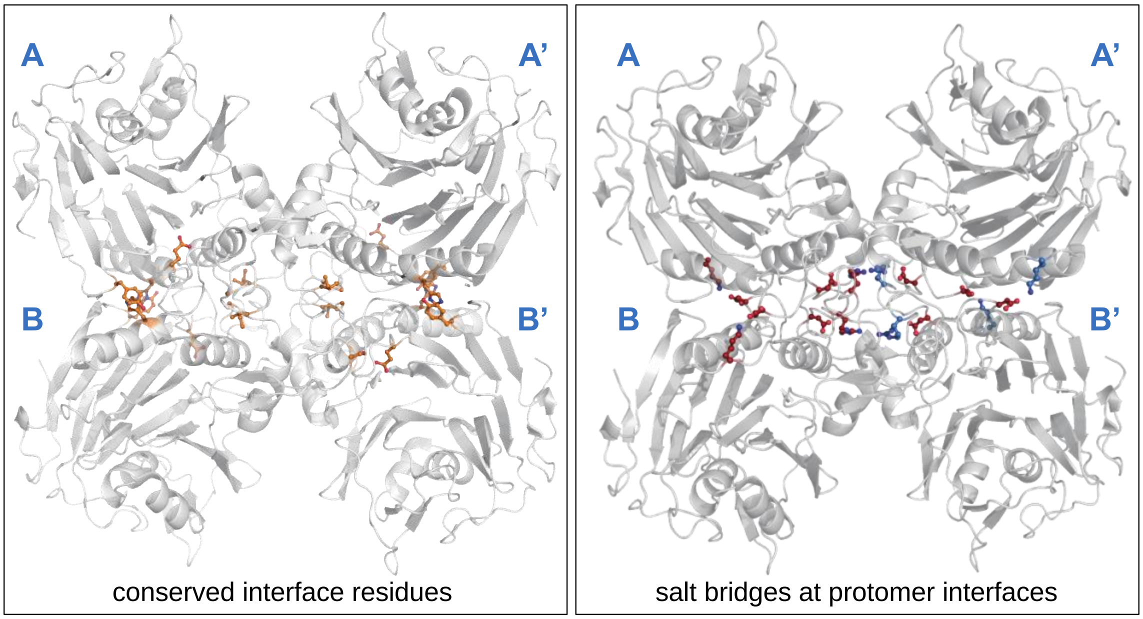

Figure S5. Conserved protomer interface residues. (Left) The BSH_{Ac} tetrameric assembly showing conserved hydrophobic and backbone residue interactions located at protomer interfaces. (Right) Amino acids involved in cross-protomer salt bridge formation. The BSH_{Ac} dimers are denoted A:B and A':B' according to established *B. longum* labeling(1).

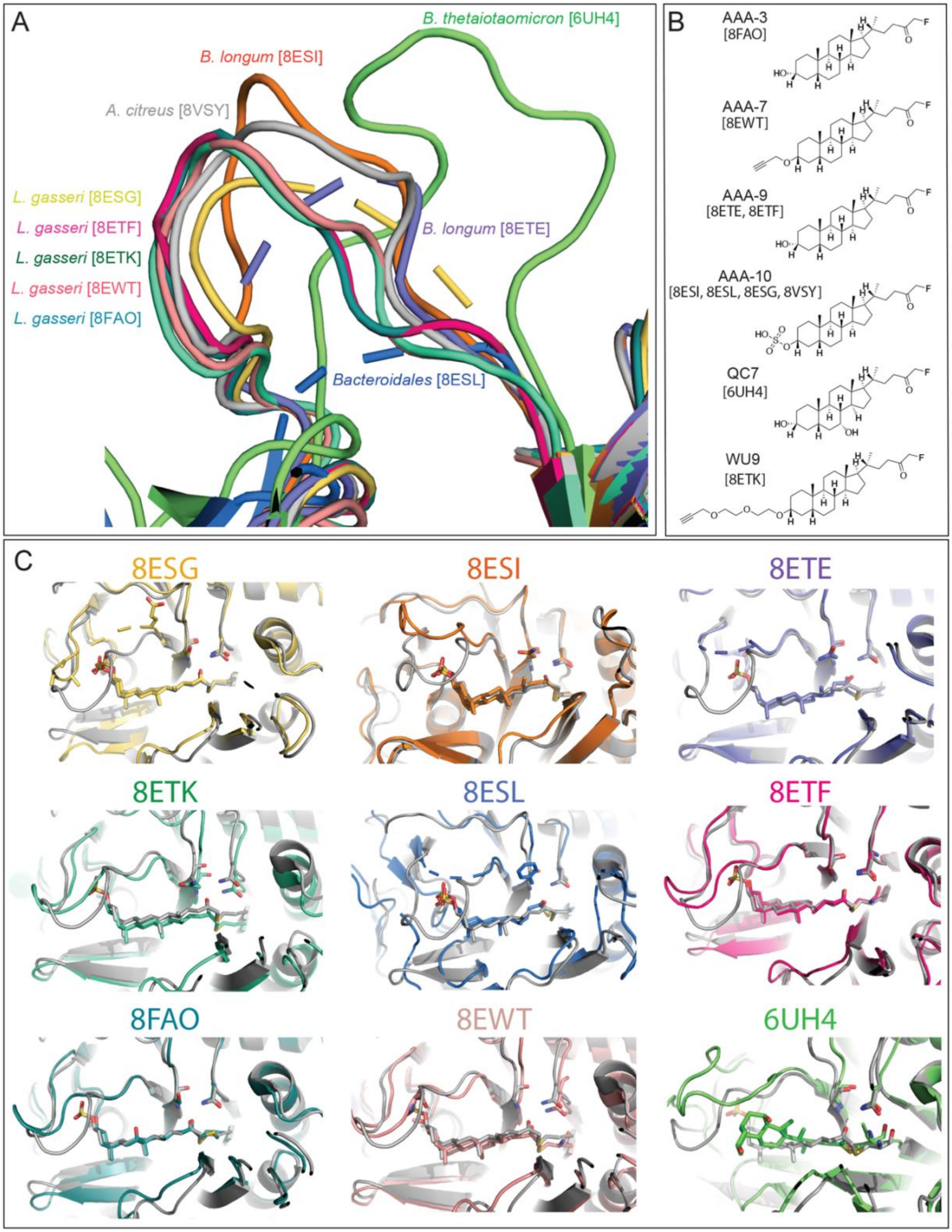

Figure S6. View of the BSH active sites upon structural superposition of BSH:inhibitor complexes. (A) Positions of the substrate binding site loop (SB loop) observed in BSH:inhibitor complex structures. For clarity, only a cartoon representation of protein is shown. **(B)** The structure of BSH inhibitors or probes that have been co-crystallized with the various BSHs shown in panels A and C. The PDB IDs are listed below the ligand or AAA series compound IDs. **(C)** Structural superpositions of the BSH_{Ac}:AAA-10 complex with other BSH:inhibitor complexes.

Table S1. Overview of reported BSH structures and BSH:inhibitor complexes

**Source Organism**	**PDB ID**	**Inhibitor/ Ligands**	**Selectivity Motif**	**Sequence ID to BSH_{Ac} (%)**	**RMSD from BSH_{Ac}**
A. citreus	8VRX		GSG		
	8VSY	AAA-10			0.33
B. longum	2HF0		GAG	57	0.7
	2HEZ				0.68
	8ESI	AAA-10			0.66
	8ETE	AAA-9			0.67
L. gasseri [BSH_A]	8ETK	WU9	GVG		0.9
	8EWT	AAA-7			0.89
	7SVG			41	0.87
	7SVF				0.89
L. gasseri [BSH_B]	8FAO	AAA-3	SRS	37	0.82
	8ETF	AAA-9			0.81
	8ESG	AAA-10			0.84
C. perfringens	7SVH		GQG	35	0.83
	2BJG				0.98
	2RLC	GLY			0.97
	2BJF	TAU, DXC			0.93
	2RF8				0.95
	2RG2				0.96
B. thetaiotaomicron	6UFY		No loop		1.0
(B. theta)	6UH4	AAA-1		22	1.1
Bacteroides spp.	8ESL	AAA-10	No loop	22	1.0
E. faecalis	5GS7		GIG	36	0.85
	6A8T				0.86
L. salivarius	8BLT	TCA	GTG	35	0.97
	8BLS	GCA			0.97
	4WL3				0.91
	5HKE				1.03
	5Y7P				0.97
L. johnsonii	7SVI		GVG	42	0.94
L. acidophilus	7SVE		SRG	35	0.97
L. ingluviei	7SVJ		SRG	38	0.94
L. reuteri	7SVK		SRG	36	0.92

Supporting Reference

1. Kumar, R. S., Brannigan, J. A., Prabhune, A. A., Pundle, A. V., Dodson, G. G., Dodson, E. J., and Suresh, C. G. (2006) Structural and functional analysis of a conjugated bile salt hydrolase from Bifidobacterium longum reveals an evolutionary relationship with penicillin V acylase. *J Biol Chem* **281**, 32516-32525
